# Multi-Objective Optimization Design of Doherty Power Amplifier Circuits Based on Non-Dominated Sorting Genetic Algorithm-II

**DOI:** 10.3390/mi17050556

**Published:** 2026-04-30

**Authors:** Hanbin Qu, Xiaopeng Zhang, Sixin Gao, Silu Yan

**Affiliations:** 1 Hebei Xinhuabei Integrated Circuit Co., Ltd., Shijiazhuang 050200, China; simbarq@sina.com (H.Q.); zhangxp@nc-ic.com (X.Z.); gaosixin2014@163.com (S.G.); 2 China Electronics Technology Group Corporation, Institute 13, Shijiazhuang 050051, China; 3 Key Laboratory for Wide Band Gap Semiconductor Materials and Devices of Education Ministry, School of Microelectronics, Xidian University, Xi’an 710071, China

**Keywords:** Doherty power amplifier (DPA), efficiency, linearity, Non-dominated Sorting Genetic Algorithm-II (NSGA-II)

## Abstract

Conventional optimization algorithms face challenges such as lengthy computation times, premature termination at non-convergent points, and the generation of local optima when addressing multi-objective optimization. A multi-objective optimization method based on the Non-dominated Sorting Genetic Algorithm-II (NSGA-II) is proposed for optimizing Doherty power amplifier circuits. The pre-layout simulation results show that, compared to traditional design methods, the optimized Doherty power amplifier circuit achieves a 6.4% increase in saturation efficiency, a 3.3% increase in 6 dB roll-off efficiency, and a 1 dB increase in saturation output power at 2.63 GHz. This approach enables multi-objective optimization design for more complex PA circuits and enhances the overall circuit performance.

## 1. Introduction

The urgent demand for ultra-high data transmission rates and ultra-wideband signals in 5G communication systems has presented traditional power amplifiers with the dual challenges of deteriorating efficiency and nonlinear distortion [1]. Doherty power amplifiers (DPAs), with their excellent average efficiency characteristics in peak-to-average power ratio signal processing, have emerged as a key circuit architecture that balances efficiency and linearity [2]. However, traditional DPA design relies heavily on engineering experience and requires extensive iterative load pull testing, nonlinear device model calibration, and circuit topology optimization, presenting significant technical bottlenecks in balancing nonlinear distortion suppression with dynamic efficiency optimization.

In recent years, many simulation and analysis tools have integrated optimization options to address complex design problems or perform fine-tuning. For example, commercial simulation software incorporates optimization algorithms such as stochastic and gradient-based methods. However, when dealing with multi-objective optimization of complex circuits involving a very large number of variables and objectives, these algorithms are prone to issues such as excessive optimization time, failure to converge, and getting stuck in local optima. To improve the efficiency of power amplifier design optimization, researchers have introduced improved optimization algorithms to assist in circuit optimization, such as the Enhanced Particle Swarm Optimization (ELPSO) algorithm [3,4], Support Vector Regression (SVR) [5], Genetic Algorithms [6], Bayesian optimization methods [7,8,9], the AmpDes automation approach [10], and the Knowledge-Assisted Synthesis Method (KASM) [11]. Nevertheless, it should be noted that satisfactory results have been achieved through design optimization for passive circuits such as filters and antennas. However, the optimization of active circuits—such as power amplifiers that require high performance or feature complex topologies—remains challenging and difficult to design [12,13]. Due to the nonlinear characteristics of active circuits, the strong nonlinearity of transistors, and the strong coupling between multiple objectives, optimization algorithms are more prone to getting stuck in local optima or converging slowly when searching for a global optimal solution.

To address the above issues, based on a combined circuit simulator and MATLAB R2024a simulation environment, a DPA circuit optimization design method using the NSGA-II algorithm is proposed, which is grounded in multi-objective optimization principles. The paper is organized as follows: Section 2 introduces the circuit design and structure of the DPA and describes the NSGA-II-based multi-objective optimization method for the DPA as well as the design optimization workflow. Section 3 applies the proposed method to the design of both single-stage PA circuits and DPA circuits, verifying the effectiveness, efficiency, and accuracy of the optimization method. Section 4 concludes the paper.

## 2. Design Methods

### 2.1. Circuit Structure

In this paper, a symmetrical high-gain Doherty power amplifier is designed based on the GaAs HBT process. The circuit consists of three amplifier stages: two driver stages and one Doherty-configuration amplifier stage. During the design process, the amplifier’s operating mode was first determined through DC simulation of the active stages. The main path was configured as Class AB, while the auxiliary path was set to Class C. Load pull techniques were employed to extract the input and output impedances of both amplifier paths, providing a basis for the design of the matching network. During the network design phase, adjustment of matching parameters is performed based on the load modulation characteristics of the carrier path and the peak path, and their cooperative operation is ensured. Optimization was conducted around core metrics such as saturation efficiency, back-off efficiency, and output power.

**Figure 1:**
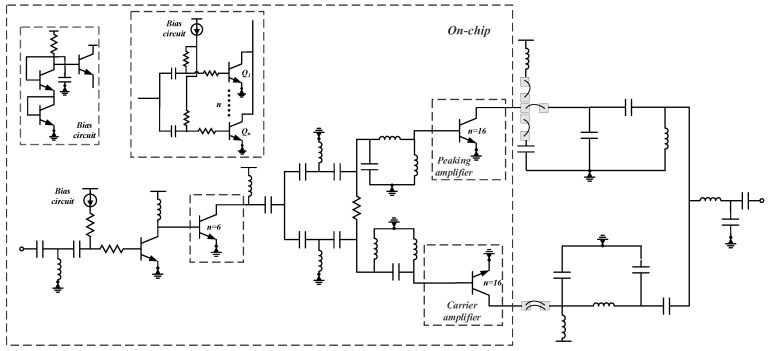
Schematic Structure of the DPA Circuit.

Specifically, in the circuit architecture, the first two driver stages employ 1 and 6 PA unit cells, respectively, operating in Class-A or Class-AB conditions. This design, which gradually increases the number of unit cells, causes the gain compression curves of the individual stages to stagger and superimpose, thereby achieving smooth amplification. Meanwhile, by adjusting the power ratio among the unit cells and the interstage matching, the gain can be flexibly distributed, ensuring that each cell operates in the linear region and suppressing nonlinear distortion. The third-stage Doherty structure adopts an equal power division strategy and consists of a two-way equal power divider, a carrier amplifier, a peaking amplifier, and a power combiner. By setting the bias points, the carrier branch is biased in deep Class-AB and the peaking branch in Class-C. Meanwhile, the capacitance and inductance in the power divider are adjusted to achieve phase compensation, thereby improving both the saturation efficiency and the back-off efficiency of the overall circuit. Specifically, by tuning the values of the reactive components in the power divider, a fixed phase offset Δφ is introduced to the input signal of the peaking path. This offset is designed to exactly cancel the inherent phase difference between the carrier and peaking paths caused by their different bias modes, such that the currents from the two paths are in phase at the output combining point. Furthermore, the design of the matching network is critical for achieving impedance matching between the main and auxiliary amplifiers, directly affecting signal transmission power, saturation efficiency, and recovery efficiency. Regarding inter-stage matching, a single choke inductor is used between the first and second stages, as well as between the second and third stages. This significantly reduces the chip area and makes the circuit more compact. The DPA schematic structure is shown in Figure 1.

### 2.2. Optimization Method

**Figure 2:**
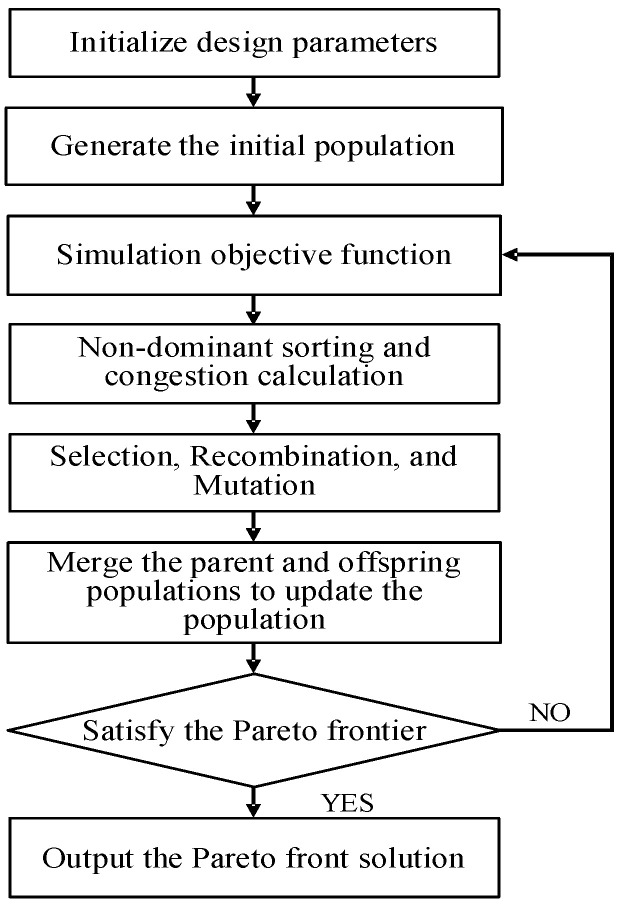
Multi-objective optimization workflow based on the NSGA-II algorithm.

As one of the most influential genetic algorithms in the field of multi-objective optimization, the NSGA-II algorithm is widely used in engineering practice [14,15,16]. Although several multi-objective evolutionary algorithms are available for analog/RF circuit optimization, NSGA-II is chosen in this work based on the following considerations. Through fast non-dominated sorting and crowding distance comparison, the NSGA-II algorithm can obtain a set of uniformly distributed Pareto-optimal solutions in a single run. This is crucial for DPA circuit design, as designers need to intuitively observe the trade-off frontier among gain, efficiency, and linearity. Moreover, the algorithm employs an elitism strategy, which ensures that superior individuals are preserved across generations. For the DPA circuit with a relatively large number of parameters, NSGA-II typically converges faster to a high-quality Pareto front. The multi-objective optimization workflow based on the NSGA-II algorithm is shown in Figure 2: First, the design parameters are initialized and an initial population is constructed, and the objective function values are calculated through simulation; next, the fitness of individuals is evaluated using non-dominance sorting and crowding index calculations. After genetic operations, offspring are generated and merged with the parent population to form a new population. This process is repeated iteratively until an optimal solution set—that is, a Pareto front solution—is obtained, ultimately achieving the optimal balance among the multiple objectives.

There are generally two approaches to optimizing PA matching networks: optimization of individual matching circuits and optimization of the entire matching circuit. Optimization of individual matching circuits involves setting the two-port impedances of the initial output (or input) matching network to the conjugate of the load impedance and 50 Ω, respectively. Subsequently, the values of the components in the matching network are adjusted to ensure that the S_11_ parameter meets the expected optimization targets, thereby achieving conjugate matching between the port and the matching network. Whole-circuit matching optimization involves establishing an initial circuit architecture. Specific values are set for multiple large-signal objectives—such as gain at the 1 dB compression point, output power (P_out_), and power added efficiency (PAE)—and different optimization weights are assigned to each objective. By combining this weighting with the objective settings, the approach enables precise, targeted optimization of the circuit’s required performance. Compared to the optimization of individual circuit matching, overall circuit matching optimization enables the coordinated optimization of the input and output matching networks. It provides greater flexibility in addressing multi-objective performance optimization problems with specific priorities, allowing for trade-offs among various performance metrics. Therefore, for a single-stage PA, its multi-objective optimization uses the errors in PAE, Gain, and P_out_ at the 1 dB compression point as the objective function, which can be expressed as follows:

(1) $$\min F(x)={\left(P_{out,t}-P_{out,s}\right)}^{2}+{\left(Gain_{t}-Gain_{s}\right)}^{2}+{\left(PAE_{t}-PAE_{s}\right)}^{2}$$

where, P_out,t_ and P_out,s_ represent the target and simulated values of P_out_, respectively; PAE_t_ and PAE_s_ represent the target and simulated values of PAE, respectively; and Gain_t_ and Gain_s_ represent the target and simulated values of Gain, respectively.

**Figure 3:**
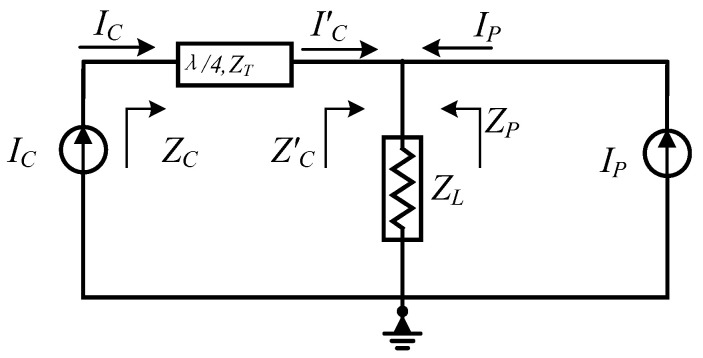
Operational diagram of the Doherty amplifier.

The multi-objective optimization of the three-stage DPA takes the errors of P_sat_, PAE_@Psat_, and PAE_@6dB BO_ as the objective functions. The optimization variables are the passive component parameters, covering the first-stage input matching network, the first-to-second interstage matching network, the second-to-third interstage matching network, the power divider, and the input and output matching networks of the final stage. The adopted sum-of-squared-errors objective function preserves the independence of each metric. Combined with the non-dominated sorting and crowding distance mechanisms of the NSGA-II algorithm, it is capable of generating a complete Pareto-optimal solution set. However, the complexity and unique characteristics of the multi-stage DPA circuit lie in the requirement to precisely realize the dynamic load modulation of the carrier and peaking paths at both the saturation and back-off points, while simultaneously ensuring phase consistency between the two signal paths at the combining point. Therefore, for the multi-objective optimization of the three-stage DPA proposed in Section 2.1, the overall framework is largely consistent with the single-stage PA optimization flow, but it is also necessary to fully exploit the characteristics of the DPA as constraints. This ensures that the optimization process consistently adheres to the fundamental principles of the DPA architecture, strictly confines the algorithmic search space to the valid operating region of the DPA, and guides the optimization algorithm to more accurately identify the optimal solutions that satisfy the performance requirements. Unlike Refs. [8,11], which indirectly improve circuit performance by optimizing intermediate-level metrics such as impedance trajectory matching or TCR network transfer characteristics, this work directly adopts the top-level performance metrics of the DPA as the optimization objectives, employs NSGA II to drive the search for matching network parameters, and generates the Pareto front, making the multi-objective trade-off relationships more intuitive. Accordingly, the objective function formulation for the three-stage DPA multi-objective optimization can be expressed as follows:

(2) $$\left\{\begin{array}{c} \min F(x)={\left(P_{sat,t}-P_{sat,s}\right)}^{2}+{\left(PAE_{@P_{sat,t}}-PAE_{@P_{sat,s}}\right)}^{2}+{\left(PAE_{@6dBBO,t}-PAE_{@6dBBO,s}\right)}^{2}\\ s.t.f_{1}(x)=|Z_{\mathrm{carrier}@\mathrm{Psat},s}-Z_{\mathrm{carrier}@\mathrm{Psat},\mathrm{ideal}}|\leq \varepsilon_{1}\\ \;\;\;f_{2}(x)=|Z_{\mathrm{peak}@\mathrm{Psat},s}-Z_{\mathrm{peak}@\mathrm{Psat},\mathrm{ideal}}|\leq \varepsilon_{2}\\ \;\;f_{3}(x)=|Z_{\mathrm{carrier}@6\mathrm{dB}\;\mathrm{BO},s}-Z_{\mathrm{carrier}@6\mathrm{dB}\;\mathrm{BO},\mathrm{ideal}}|\leq \varepsilon_{3}\\ \;\;f_{4}(x)=|Z_{\mathrm{carrier}@6\mathrm{dB}\;\mathrm{BO},\mathrm{ideal}}/Z_{\mathrm{peak}@6\mathrm{dB}\;\mathrm{BO},s}|\leq \varepsilon_{4}\\ \;\;f_{5}(x)=|\varphi_{\mathrm{carrier}@\mathrm{Psat},s}-\varphi_{\mathrm{peak}@\mathrm{Psat},s}|\leq \varepsilon_{5} \end{array}\right.$$

where P_sat,t_ and P_sat,s_ represent the target value and simulated value of P_sat_, respectively; PAE_@Psat,t_ and PAE_@Psat,s_ represent the target value and simulated value of PAE_@Psat_, respectively; PAE_@6dB BO,t_ and PAE_@6dB BO,s_ represent the target value and simulated value of PAE_@6dB BO_, respectively. Functions *f*_1_(x) to *f*_5_(x) are all constraint conditions of the multi-objective optimization. *Z*_carrier@ Psat,s_ and *Z*_carrier@ Psat,ideal_ represent the simulated and ideal impedance values at the combining point for the carrier path at saturation, respectively; *Z*_carrier@6dB BO,s_ and *Z*_carrier@6dB BO,ideal_ represent those for the carrier path at 6 dB back-off, respectively; *Z*_peak@ Psat,s_ and *Z*_peak@ Psat,ideal_ represent the simulated and ideal impedance values at the combining point for the peaking path at saturation, respectively; *Z*_peak@6dB BO,s_ represents simulated impedance value at the combining point for the peaking path at 6 dB back-off. φ_carrier@ Psat,s_ represents the simulated current phase at the junction when the carrier path is saturated, and φ_peak@ Psat,s_ represents the simulated current phase at the junction when the peak path is saturated. *ε*_1_ to *ε*_5_ are the engineering tolerance thresholds, all of which are set to 5% in this work. It should be noted that *Z*_carrier@ Psat,ideal_ and *Z*_peak@ Psat,ideal_ are set to 50 Ω, while *Z*_carrier@6dB BO,ideal_ is set to 25 Ω, as derived from the classical symmetrical DPA load modulation theory. Figure 3 shows an operational diagram to analyze the Doherty amplifier circuit. The load impedance relationships for the carrier and peaking paths are given by Equations (3) and (4) [17].

(3) $$Z_{C}=\left\{\begin{array}{c} \frac{Z_{T}^{2}}{Z_{L}},0<\nu_{in}<V_{in,\max}/2\\ \frac{Z_{T}^{2}}{Z_{L}(1+I_{C}/I_{P})},V_{in,\max}/2<\nu_{in}<V_{in,\max} \end{array}\right.$$

(4) $$Z_{P}=\left\{\begin{array}{c} \infty ,0<\nu_{in}<V_{in,\max}/2\\ Z_{L}(1+I_{C}/I_{P}),V_{in,\max}/2<\nu_{in}<V_{in,\max} \end{array}\right.$$

where Z_L_ is the load impedance of the Doherty amplifier; I_C_ and I_P_ represent the fundamental currents of the carrier amplifier and the peaking amplifier, respectively; and Z_C_ and Z_P_ are the output load impedances of the carrier amplifier and the peaking amplifier, respectively. For the classical symmetrical DPA, Z_T_ is set to 50 Ω and Z_L_ is set to 25 Ω (Z_opt_ = 50 Ω, Z_L_ = 1/2 × Z_opt_). Taking the combining point as the reference plane, Z_C_′ is calculated. At saturation, I_P_ = I_C_. Substituting into Equation (1) yields Z_C_ = 50 Ω and Z_C_′ = (Z_T_)^2^/Z_C_ =50 Ω. Substituting into Equation (2) yields Z_P_ = 50 Ω. At 6 dB back-off, substituting into Equation (1) yields Z_C_ = 100 Ω and Z_C_′ = (Z_T_)^2^/Z_C_ = 25 Ω.

### 2.3. Co-Simulation Environment

**Figure 4:**
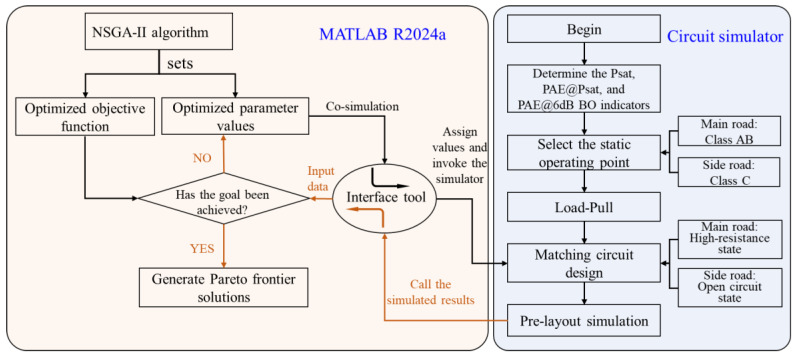
DPA multi-objective optimization workflow based on the co-simulation environment.

Implementing multi-objective circuit optimization using the NSGA-II algorithm requires the circuit simulator and MATLAB co-simulation environment, as illustrated in Figure 4. In this co-simulation environment, MATLAB serves as the algorithm implementation platform, where NSGA-II algorithm parameters are configured and optimization objectives are defined. Component parameters are then passed to the circuit simulator via an interface to perform circuit schematic simulation. After simulation, a results file containing key metrics such as PAE, Gain, and P_out_ is generated. MATLAB reads and evaluates the multi-objective optimization function. If the preset objectives are not met, parameters are automatically adjusted to enter the next iteration; if a Pareto optimal solution set is obtained, the final results are outputted. Using the optimized results, the layout is subsequently drawn and post-layout simulation is performed. This methodology achieves seamless integration between algorithmic optimization and circuit schematic simulation, significantly improving design efficiency through automated iteration.

## 3. Results and Discussion

**Figure 5:**
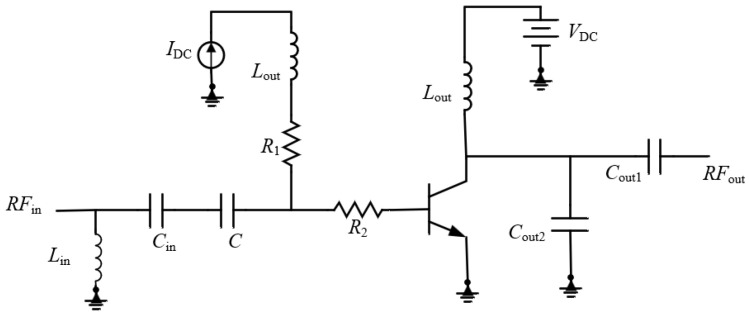
Single-Stage Power Amplifier Circuit.

This paper first verifies the effectiveness of the proposed method using a single-stage PA circuit. The circuit schematic is shown in Figure 5. This circuit uses a load/source extraction method to obtain the transistor impedance, and the initial values of the input and output matching components are selected based on conjugate matching of the extracted values. The values of *L*_in_, *C*_in_, *C*_out1_, *C*_out2_, and *L*_out_ are written into the Match.mdf file generated by MATLAB and called via the interface tool. The variable tool is used to assign these component values to corresponding variables in the circuit, and the interface tool in MATLAB is used to call the circuit simulator for automatic simulation, thereby obtaining the initial circuit performance. The circuit’s 1 dB compression point, P_out_, Gain, and PAE are 14.7 dBm, 14.7 dB, and 20.339%, respectively.

**Figure 6:**
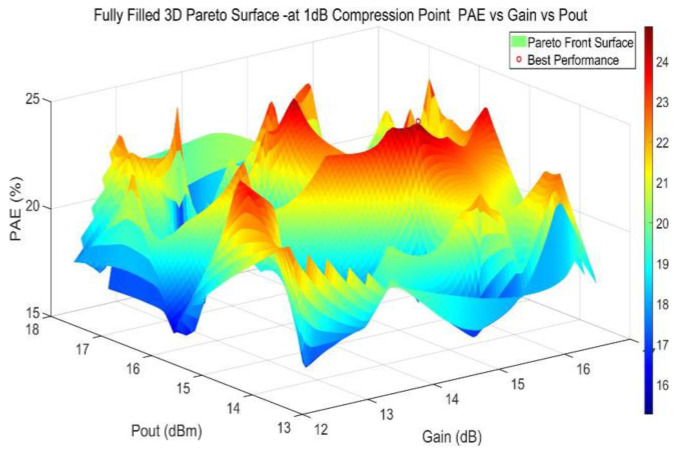
Pareto front solution generated using MATLAB.

The NSGA-II algorithm was configured with a population size of 50, a maximum of 100 generations, a crossover probability of 0.8, and a mutation probability of 0.1. For unbiased multi-objective optimization, all objective function weights were set equal. The capacitance and inductance values of the five passive components in the matching network were allowed to vary from 50% to 150% of their initial values. Based on the optimization algorithm and simulation platform proposed in Section 2.3, Figure 6 shows the Pareto front solution for the joint circuit optimization. The optimization results show that the 1 dB compression point (P_out_), Gain, and PAE of the circuit are 15.3 dBm, 15.3 dB, and 24.9%, respectively. A comparison reveals that these results are consistent with the circuit-simulated results, validating the feasibility of the joint simulation platform. Compared to the pre-optimization state, both P_out_ and gain have improved by 0.6 dB, and PAE has increased by 4.6%, effectively achieving the optimization of the circuit’s multi-objective performance and meeting the expected design goals.

To verify the effectiveness and efficiency of the NSGA-II algorithm, this paper compared it with several optimization algorithms (random algorithm, gradient algorithm, and genetic algorithm) in optimizing the initial circuit under the same objective. The random algorithm and the gradient-based algorithm were set with a maximum of 10,000 and 100 iterations, respectively. The genetic algorithm was configured with a population size of 50, a maximum of 100 iterations, a crossover probability of 0.8, a mutation probability of 0.1, and an elitism retention ratio of 0.2. All algorithms were independently executed 10 times on the same hardware platform (Intel Core i9-13900K @ 5.8 GHz single-core turbo, 64 GB DDR5-6400, Windows 11 Pro 23H2). The results show that while the random algorithm achieved some optimization in PAE performance, it did so at the cost of significantly reducing P_out_ and Gain performance. In contrast, the NSGA-II algorithm and the gradient algorithm demonstrated clear advantages over other algorithms in terms of P_out_ and Gain performance at the 1 dB compression point, while the NSGA-II algorithm performed most notably in improving PAE performance compared to other algorithms. Table 1 details the simulation time consumed by each optimization algorithm upon convergence. The clock starts when the algorithm parameters are initialized and stops when the last convergence check is satisfied, and the reported values are averaged over multiple complete co-simulation runs. The results indicate that, under the original unified objective function, neither the gradient-based algorithm nor the genetic algorithm was able to reach the convergence threshold within the maximum number of iterations; the core metrics of their final solutions all exhibited errors exceeding 10%. Feasible solutions that satisfied the predefined objectives and reached the convergence threshold could only be obtained after reducing the optimization objectives. The random algorithm required a simulation time an order of magnitude higher than the other algorithms upon convergence. The results indicate that, compared to other optimization algorithms, the NSGA-II algorithm demonstrates significant advantages in the multi-objective optimization process, achieving superior performance metrics in a shorter time, thereby reflecting its efficiency and superiority in circuit multi-objective optimization problems.

**Figure 7:**
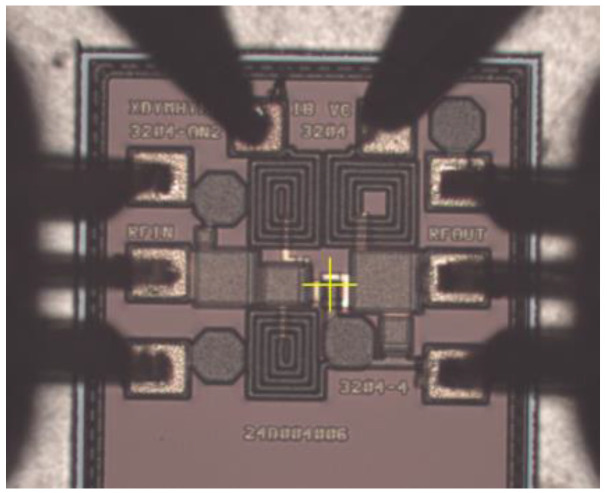
Microscopic image of the circuit under testing.

**Figure 8:**
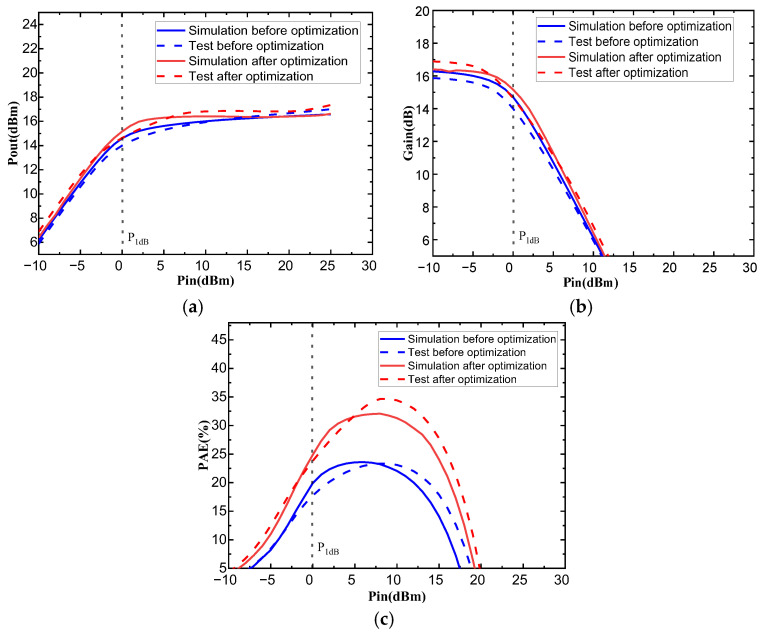
Comparison of simulation and test performance before and after circuit optimization: (**a**) Comparison of P_out_ performance before and after optimization; (**b**) Comparison of gain performance before and after optimization; (**c**) Comparison of PAE performance before and after optimization.

To further validate the optimization method employed, this paper conducted layout design and wafer fabrication tests on both the initial circuit and the circuit optimized using the NSGA-II algorithm. Figure 7 shows the on-wafer test micrograph of the circuit, and Figure 8 compares the simulated and measured performance of the circuit before and after optimization. The results indicate that the optimized circuit achieved significant improvements in all performance metrics. The measured P_out_ and Gain at the 1 dB compression point of the optimized circuit both increased by 0.63 dB compared to the pre-optimized circuit, and the PAE improved by 6.06%, strongly demonstrating the effectiveness and accuracy of the optimization method adopted in this paper.

**Figure 9:**
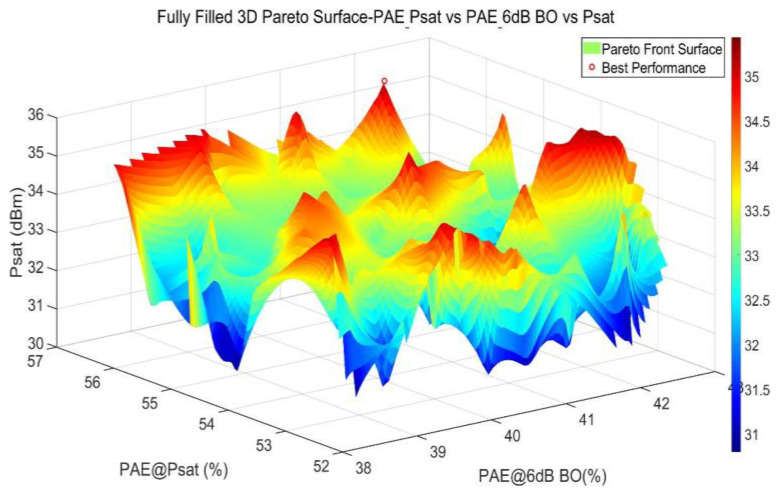
Pareto-optimal solutions for third-order DPA.

In the multi-objective optimization design of a three-stage DPA, the parameter values of all matching components are written into the Match.mdf file generated by MATLAB and called via the interface tool. These passive matching components include the first-stage input matching component, the matching components between the first and second stages and between the second and third stages, the power divider component, and the third-stage input and output matching components. Among these, the matching circuit elements in the first two stages of the three-stage DPA must be dynamically selected based on changes in the input impedance of the subsequent stage, and different values for these elements have a significant impact on the DPA’s P_sat_ performance. The power divider element is responsible for precisely controlling the power distribution ratio and phase characteristics of the two output paths in the final stage, ensuring strict phase coherence between the two signals during power combining. The matching circuit components in the final stage not only affect the individual performance of the two paths but also directly determine the accuracy of dynamic load modulation and the power roll-off range when the paths are combined, thereby influencing the overall performance between the saturation point and the roll-off point. During the optimization process, we used variable tools to assign these component values one-to-one with the variables in the circuit and called the circuit simulator via the interface tool in MATLAB for automatic simulation. After the ADS-MATLAB co-optimization process, MATLAB outputs the Pareto front solutions for the three-stage DPA as shown in Figure 9. The optimization results indicate that, at a P_sat_ of 35.439 dBm, the design achieves a PAE_@Psat_ of 55.612% and a PAE_@6 dB BO_ of 41.772%.

**Figure 10:**
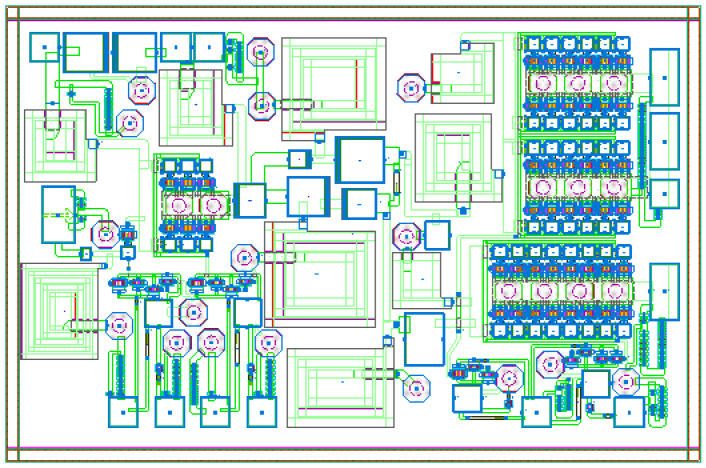
Optimized three-level DPA layout.

**Figure 11:**
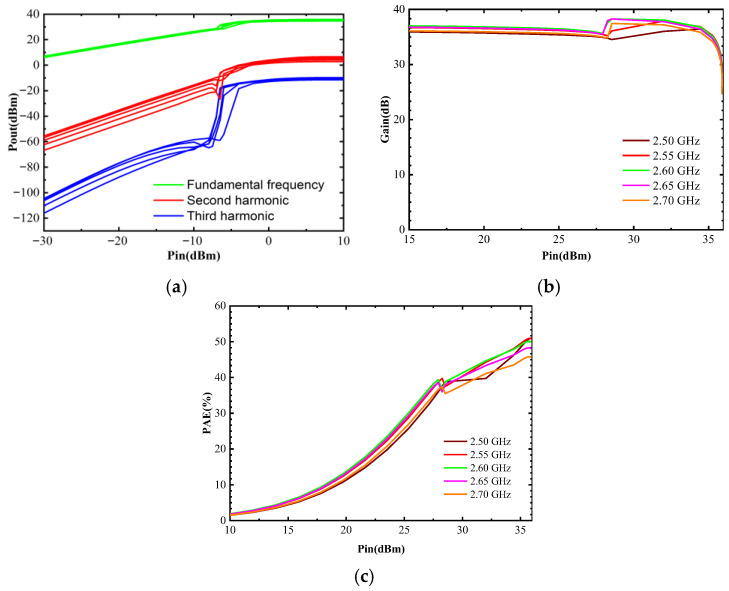
Post-layout simulation of the large-signal performance of the three-stage DPA circuit. (**a**) simulated results of the power performance; (**b**) simulated results of the Gain performance; (**c**) simulated results of the PAE performance.

**Figure 12:**
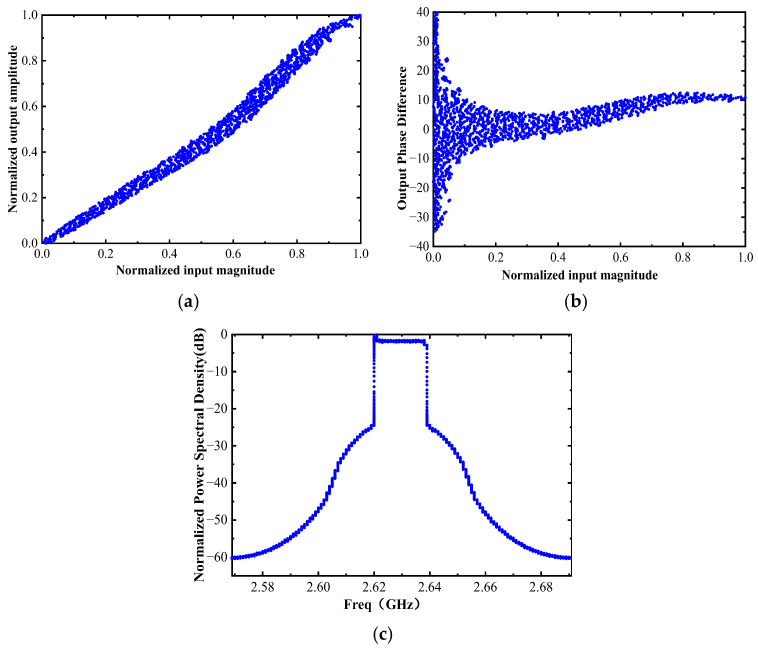
Simulated AM/AM, AM/PM, and PSD of the Optimized DPA. (**a**) AM/AM; (**b**) AM/PM; (**c**) PSD.

Figure 10 and Figure 11 show the layout of the optimized DPA circuit and its post-layout simulation performance, respectively. Post-layout simulations indicate that the circuit can achieve a saturation PAE exceeding 47.8% within the target frequency band and a back-off PAE higher than 34%, suggesting favorable power back-off characteristics under nominal simulation conditions. Meanwhile, the circuit’s large-signal gain is stable at around 36 dB, and P_sat_ consistently exceeds 35 dB, indicating competitive output power capability in post-layout simulation. Figure 12 shows the simulated amplitude modulation/amplitude modulation (AM/AM), amplitude modulation/phase modulation (AM/PM), and power spectral density (PSD) characteristics of the DPA circuit. The adjacent channel power ratio (ACPR) for the left and right adjacent channels of the main channel is −28.20 dBc and −28.28 dBc, respectively.

Table 2 compares the performance data before and after optimization, with the small-signal simulation frequency range set at 2.48–2.68 GHz and the large-signal simulation conducted at 2.63 GHz. The pre-optimization simulation results show that P_sat_ increased from 34.4 dBm to 35.6 dBm, and PAE_@6dB BO_ improved from 38.4% to 41.7%. Although the post-optimization simulation results showed slight declines in certain metrics, the overall performance still outperformed the pre-optimization circuit’s pre-layout simulation data. Regarding small-signal parameters, simulated S_22_ of the optimized design was significantly reduced to below −18 dB in the pre-layout simulation, indicating that the output matching was effectively enhanced at the schematic level. Although S_21_ decreased slightly, harmonic distortion was significantly reduced: second-order harmonic distortion dropped from −58 dBc to −63 dBc, and third-order harmonic distortion decreased from −68 dBc to −74 dBc in post-layout simulation. These results indicate that the optimization algorithm contributed to improving the circuit’s linearity in simulation to some extent while enhancing the target performance. Table 3 compares the post-layout simulation results of the DPA optimized in this work with the measured performance of other reported 2.6 GHz DPAs. While certain individual performance metrics may not exceed the best values reported for other 2.6 GHz DPAs, driven by the NSGA-II-based automated flow, the entire process—from design space exploration to Pareto front generation—can be completed within a few hours, which is significantly more efficient than the conventional manual design approach. Overall, the optimized DPA exhibits significant improvements in power output and efficiency compared to the initial schematic design, meeting the expected design objectives.

## 4. Conclusions

A method based on the NSGA-II algorithm and multi-objective optimization principles for the optimized design of DPA power amplifiers is proposed in this paper. This method performs multi-objective optimization of the circuit’s saturated output power, saturated output efficiency, and cut-back output efficiency by adjusting the values of each component in the matching network as optimization parameters. This algorithm was applied to single-stage PA and multi-stage DPA designs. For the single-tube, single-stage PA, multi-objective optimization was performed on P_out_, Gain, and PAE at the 1 dB compression point, yielding improvements of 0.6 dB, 0.6 dB, and 4.3%, respectively, which were verified through chip fabrication. Subsequently, multi-objective optimization was performed on the more complex multi-transistor three-stage DPA. The schematic simulation results show that, compared with the initial design, P_sat_, PAE_@Psat_, and PAE_@6dB BO_ increased by 1 dB, 6.4%, and 3.3%, respectively. These results further demonstrate the efficiency and applicability of the NSGA-II algorithm in the multi-objective optimization design of complex PA architectures.

## Figures and Tables

**Table 1:** Comparison of Convergence and Simulation Time for Various Optimization Algorithms.

Optimization Algorithms	Convergence (Convergence Criterion)	Convergence Simulation Time (s)	Standard Deviation
NSGA-II	Convergence (the Pareto front remains unchanged for 10 consecutive generations)	263	18.7
Random algorithms	Convergence (no reduction in the objective function was observed beyond 5000 iterations)	3210	245.3
Gradient algorithm	Convergence after reducing the optimization objective (the relative change in the objective function < 1 × 10^−4^)	456	32.1
Genetic Algorithms	Convergence after reducing the optimization objective (the elite individual remained unchanged for 10 successive generations)	366	27.9

**Table 2:** Comparison of Performance Data Before and After Optimizations.

Performance Specifications	Before	After	
	Pre-Layout Simulation	Pre-Layout Simulation	Post-Layout Simulation
**S_21_(dB)**	≥45	≥40	≥35
**S_11_(dB)**	≤−12	≤−12	≤−11
**S_22_(dB)**	≤−14	≤−18	≤−10
**S_12_(dB)**	≤−50	≤−65	≤−58
**Gain (dB) _@6dB BO_**	45	40	36.5
**P_sat_ (dBm)**	34.4	35.6	35.1
**2^nd^ harmonic (dBc) _@6dB BO_**	−58	−63	−55
**3^rd^ harmonic (dBc) _@6dB BO_**	−68	−74	−65
**PAE (%) _@6dB BO_**	38.4	41.7	34.9
**ICCQ (mA)**	116	109.9	112

**Table 3:** Performances Summary and Compare with Other DPAs.

References	Technology	Freq. (GHz)	Psat (dBm)	Gain (dB)	PAE (%)
[18]	GaN	2.655	—	30.9	41 (PAE_@6dB BO_)
[19]	GaAs	2.6	~28.5	—	50 (PAE_@8.5dB BO_)
[20]	LDMOS MMIC	2.5~2.7	40	30	50 (PAE_@7.5dB BO_)
This work *	GaAs	2.63	35.1 *	36.5 *	34.9 * (PAE_@6dB BO_)

* simulation results.

## Data Availability

Data are contained within the article.
